# Response to “How can WhatsApp facilitate the future of medical education and clinical practice?”

**DOI:** 10.1186/s12909-020-02455-0

**Published:** 2021-01-14

**Authors:** Enda O’Connor, Eamonn Coleman

**Affiliations:** University of Dublin Trinity College, Dublin, Ireland

Dear Editor

We thank the authors for their correspondence related to our scoping review [1]. They propose expanding the use of an instant messaging application (IMA) in the undergraduate setting, specifically for problem-based learning (PBL) and during clinical student clerkships.

The authors state some orthodoxies about undergraduate medical learning – that it is opportunistic, experiential and is effective when it occurs in groups – before asserting that WA can enhance each of these elements of learning. We are in agreement with this. A majority of published literature suggests that, concerns about intrusiveness notwithstanding, WA makes learning more convenient, collaborative, inclusive and enjoyable. It promotes group learning, and enhances efficiency by streamlining the organisational aspects of learning. Importantly, it is also an effective educational tool. In our review, 9 studies evaluated the IMA in an undergraduate setting [2–10], 8 of which, including 3 randomised controlled trials [3, 4, 9] demonstrated a learning benefit.

If the authors hope to expand the role of an IMA in undergraduate education, further analysis of these 9 studies reveals some useful insights to guide their planning. Eight used an online moderator/facilitator and 7 used WA in a blended model of learning. Furthermore, 6 used the IMA in discrete learning modules rather than for broad curricular learning, all of which centred around basic science topics (histology, bacteriology, pharmacology, anatomy, virology and physiology). Current evidence therefore favours WA use for discrete topical blended learning, guided by a moderator, and remote from the bedside. This is not to state that it is of no value in undergraduate clinical learning; just that the current published research does not support its use in this setting.

The authors relate WhatsApp specifically to PBL and to clinical attachments. We draw their attention to a qualitative study included in our review by Raiman et al. evaluating the IMA in these settings [5]. This small study yields interesting insights into IMA learning, in particular the categorisation of the WA content into educational, organisational and social messages. Users also lamented the lack of face-to-face interaction with IMA learning. Decisions about the role of WA in learning activities therefore need to take account of how the platform will be used and what messages will be promoted therein.

The principal assertion of the authors that the role of WA should be more widely integrated into undergraduate medical education activities is a laudable one. Consistent with our instructional design model however, the objectives of WA as an educational tool need to be clearly articulated prior to the design and delivery of a new intervention [1]. Assuming that the purpose of the WA group is primarily educational, the following guiding questions may be useful to a new IMA education designer.

1. Will WA be used as a standalone or blended learning tool? As noted above, the strongest evidence supports a blended model of learning.
2. Will the discourse on the IMA be solely educational or will organisational and/or social messages be permitted? This centres on whether the core learning occurs through WA discussions, or whether WA is used to facilitate learning occurring elsewhere. For the former, online activities should be clearly mapped to predetermined learning objectives.
3. Will WA learning use a pre-defined curriculum or not? It appears to be more suited to discrete quanta of learning than to large curricula.
4. Will learner participation be voluntary or mandatory?
5. Will a moderator be required and has he/she experience in this online role? WA groups used primarily for educational purposes, especially those where core learning occurs through the online discussions, require a trained moderator.
6. Will the WA group discussion be used as a summative student assessment tool? Few data exist regarding the use of WA for formal undergraduate assessment [11]. Measuring participation in online discussions ignores the fact that effective passive learning can occur in an IMA group. Furthermore, active participation may be hindered by reticence and unfamiliarity with or poor access to the platform. Finally, problems posed on a WA group can effectively only be “answered” once, the solution thereafter visible to all group members. We call this phenomenon the “answer exposure”, a shortcoming which denies some learners the opportunity to demonstrate their knowledge [8]. In our experience however, the IMA environment is indeed useful for formative assessment, clarifying errors and misunderstandings in a transparent and collaborative environment.

Finally, the authors correctly assert that the COVID-19 pandemic has inspired a reappraisal of the role of technology-enhance learning (TEL) in medical education. Numerous reports highlight a plethora of eLearning strategies, described as a “medical education revolution” [12]. These strategies include learner management systems (Blackboard®, Fry-it®), videoconferencing (Zoom®, Teams®), lecture recording platforms (Panopto®), digital clinical placements and eSimulation strategies [12]. The additional appeal of using an IMA for information-sharing in an era of physical distancing seems obvious. Based on the paucity of medical education articles related to COVID-19 and IMA learning however [13], it is possible that the pandemic has paradoxically driven TEL away from WA and towards more formal online learning tools. Of note, we also caution against overuse of mobile internet devices in an era of electronic patient records. The obvious concern is the perception of a doctor or medical student interacting more with their electronic devices than with the patient.

In summary, WhatsApp is a cheap, accessible learning resource which the authors suggest requires further integration into undergraduate education. In principle, we support an expansion of blended learning strategies in medical education but caution however against the use of an IMA without theory-driven instructional design considerations. We also encourage reflection on the use of other more formal and adaptable technologies to enhance learning. We encourage the authors to consider the use of our instructional design model to integrate IMA education into their practice.
